# Pseudomyxoma peritonei of a mature ovarian teratoma caused by mismatch repair deficiency in a patient with Lynch syndrome: a case report

**DOI:** 10.1186/s12881-016-0356-5

**Published:** 2016-12-09

**Authors:** Yoshimasa Gohda, Rei Noguchi, Tomoko Horie, Toru Igari, Harumi Nakamura, Yasunori Ohta, Kiyoshi Yamaguchi, Tsuneo Ikenoue, Seira Hatakeyama, Nozomi Yusa, Yoichi Furukawa, Hideaki Yano

**Affiliations:** 1 Division of Colorectal Surgery, Department of Surgery, National Center for Global Health and Medicine, 1-21-1 Toyama, Shinjuku-ku, 162-8655 Tokyo, Japan; 2 Division of Clinical Genome Research, Institute of Medical Science, The University of Tokyo, 4-6-1 Shirokanedai, Minato-ku, 108-8639 Tokyo, Japan; 3 Pathology Division of Clinical Laboratory, National Center for Global Health and Medicine, 1-21-1 Toyama, Shinjuku-ku, 162-8655 Tokyo, Japan; 4 Department of Pathology, Research Hospital, Institute of Medical Science, The University of Tokyo, 4-6-1 Shirokanedai, Minato-ku, 108-8639 Tokyo, Japan; 5 Department of Applied Genomics, Research Hospital, Institute of Medical Science, The University of Tokyo, 4-6-1 Shirokanedai, Minato-ku, 108-8639 Tokyo, Japan; 6 Division of Clinical Genome Research, Advanced Clinical Research Center, The Institute of Medical Science, The University of Tokyo, 4-6-1 Shirokanedai, Minato-ku, 108-8639 Tokyo, Japan

**Keywords:** Pseudomyxoma peritonei, Ovarian teratoma, Lynch syndrome, Mismatch repair, Microsatellite instability

## Abstract

**Background:**

Pseudomyxoma peritonei (PMP) is a rare disease with an estimated incidence of 1–2 cases per million individuals per year. PMP is characterized by the accumulation of abundant mucinous or gelatinous fluid derived from disseminated tumorous cells. Most of the tumorous cells are originated from rupture of appendiceal neoplasms, but some are from the metastasis of cancer of the colon, ovary, fallopian tube, urachus, colorectum, gallbladder, stomach, pancreas, lung and breast. Although frequent mutations in *KRAS* and/or *GNAS* genes have been reported, precise molecular mechanism underlying PMP remains to be elucidated. It is of note that mucinous tumour is one of the frequent histological features of colorectal cancer (CRC) in Lynch syndrome (LS), an autosomal dominantly inherited disease caused by a germline mutation of the DNA mismatch repair (MMR) genes including human mutL homolog 1 (*MLH1*), human mutS homolog 2 (*MSH2*), human mutS homolog 6 (*MSH6*), and postmeiotic segregation increased 2 (*PMS2*). Therefore, typical LS-associated tumours show mismatch repair instability. Although LS patients are most strongly predisposed to CRC, PMPs from mucinous CRC have not been reported in LS patients.

**Case presentation:**

In this report, we report a case of PMP originating from an ovarian teratoma in a LS patient. The patient had surgical treatment of PMP arising from an ovarian teratoma at the age of 38 years, and later developed a transverse colon cancer at the age of 40. The patient’s family history fulfilled the Amsterdam criteria, and genetic analysis of the peripheral leukocytes identified a germ line mutation in the *MLH1* gene (*MLH1* c.1546dupC p.Q516PfsX3). Interestingly, immunohistochemical staining showed that the expression of MLH1 was lost in the colon cancer as well as the ovarian teratoma. Consistent with the loss of MLH1 expression, both tumours showed high microsatellite instability (MSI-H).

**Conclusion:**

This case suggested that LS patients may develop various types of tumours including ovarian PMP, and that mismatch repair deficiency may play a role in the development of PMP derived from, at least, a part of ovarian teratomas.

## Background

Pseudomyxoma peritonei (PMP) is a very rare disorder, and a nationwide study in Netherland disclosed that the estimated incidence of PMP is 1–2 cases per million individuals per year [1]. The condition is characterized by the accumulation of abundant mucinous or gelatinous fluid that is produced from tumorous cells disseminated in the abdominal cavity and pelvis [2]. Patients with PMP usually suffer from abdominal distension, change of body weight, abdominal or pelvic pain, and/or digestive disorder by the accumulation of ascites and/or the progression of disseminated lesion. Tumour cells of PMP are most frequently derived from rupture of appendiceal neoplasms, but occasionally from the tumours in other organs including the ovary [1, 3], fallopian tube [4], urachus [1, 5], colorectum [1, 6], gallbladder [7], stomach [8], pancreas [1, 9], lung [10] and breast [11]. The average onset of the disease is reportedly from 49 to 62 years old, which is earlier than other abdominal malignancies [1, 12]. Several molecular studies have disclosed that frequent mutations in *KRAS* and *GNAS* are involved in appendiceal PMP [13–18]. However precise molecular mechanism of PMP remains to be elucidated.

Regarding the treatment of PMP, cytoreductive surgery (CRS) combined with hyperthermic intraperitoneal chemotherapy (HIPEC) has greatly improved the prognosis of patients. A recent study of 1000 appendiceal PMPs revealed that 738 patients treated with complete CRS and HIPEC showed 87.4% of 5-year and 70.3% of 10-year survival [19]. However, little is known about the prognosis of PMP originated from ovarian teratoma.

Lynch syndrome (LS) or hereditary non-polyposis colorectal cancer (HNPCC) is an autosomal dominantly inherited syndrome accounting for 2–7% of all colorectal cancers (CRC) [20, 21]. It is caused by a germline mutation of the DNA mismatch repair genes (MMR) including *human mutL homolog 1* (*MLH1*), *human mutS homolog 2* (*MSH2*), and to a lesser extent, *human mutS homolog 6* (*MSH6*), and *postmeiotic segregation increased 2* (*PMS2*). LS is characterized by the susceptibilities for various LS-associated tumours such as colorectal, endometrial, stomach, small bowel, ovarian, urothelial, bladder, and biliary tract cancers [22, 23]. Ovarian cancer is one of Lynch syndrome-related extra-colorectal neoplasms and the life time risks are 4–12%. The mean age at diagnosis of the ovarian cancers is 42.5 years and approximately 30% of the cancers is diagnosed before the age of 40 years [24]. The pathology type of Lynch syndrome-related ovarian cancers is similar to sporadic ovarian cancers including mostly epithelial cell-derived tumours. Even in sporadic ovarian cancers, 3–8% of ovarian mucinous tumours occur from mature cystic teratoma which indicates that teratoma is a very rare form of ovarian tumours [25]. Although the frequency of mucinous colorectal cancer and that of ovarian tumours are higher in the LS patients compared with normal population, PMP has never been reported in LS patients. Most patients with Lynch syndrome are clinically identified by the revised Amsterdam’s criteria, or the revised Bethesda’s guideline for the test of microsatellite instability. The tumours associated with LS patients commonly show microsatellite instability, a hallmark of mismatch repair deficiency. In addition to testing for microsatellite instability, immunohistochemical staining of responsible gene products is often used for the screening of the deficiency.

Here, we report for the first time, a case of ovarian PMP in a patient with Lynch syndrome, and show the association of the ovarian tumour with mismatch repair deficiency.

## Case presentation

**Fig. 1:**
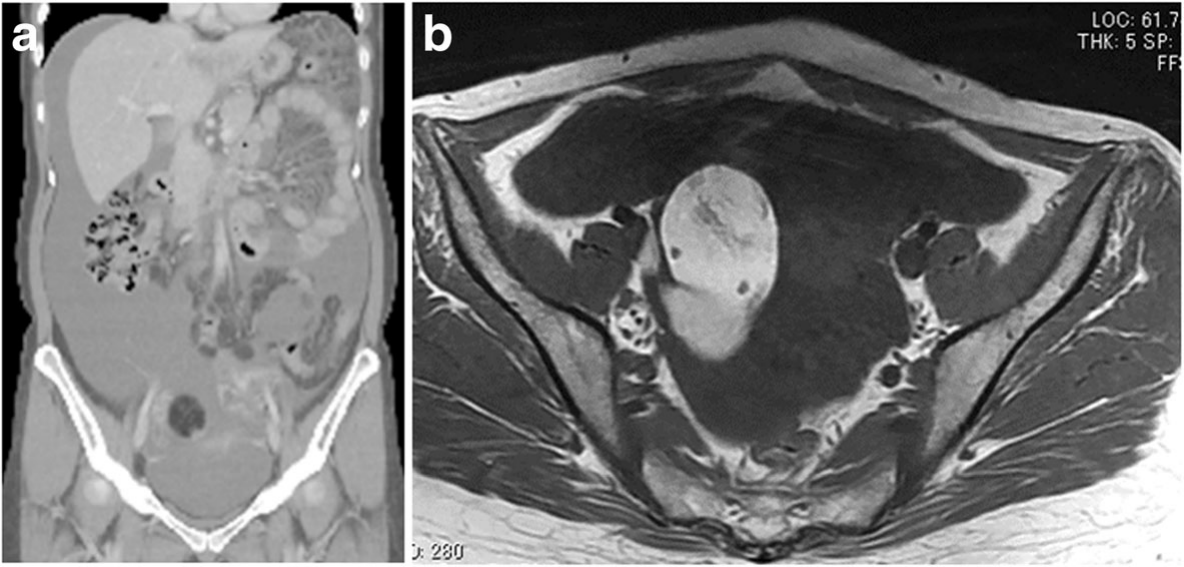
**a** Contrasted computed tomography showed distended abdominal cavity filled with low-density fluid. **b** A pelvic magnetic resonance image of the right ovarian mass with high signal intensity on T2-weighted image

A 38 year-old woman presented with progressive abdominal distension. Contrasted abdomino-pelvic CT revealed that the distended abdominal cavity of the patient was filled with low-density fluid, and showed a degree of scalloping sign on the liver surface (Fig. 1a). A 7-cm sized multi-locular cystic mass containing fat tissue, which was thought to be originating from the right ovary, was identified in the pelvis. However, the patient’s appendix and left ovary appeared normal. Pelvic magnetic resonance image showed cystic component with high signal intensity on T1- and T2-weighted images, and fat-saturated T1-weighted images showed a drop in the signal intensity of the right ovarian mass (Fig. 1b). These imaging studies suggested a teratoma of the right ovary resulting in pseudomyxoma peritonei. Colonoscopy and gastroscopy were normal. The laboratory test of tumour markers showed that CEA, CA19-9, and CA125 were elevated to 15.5 ng/ml (Upper normal limit [UNL] < 5 ng/ml), 59.8 U/ml (UNL < 37 U/ml), and 39.7 U/ml (UNL < 35 U/ml), respectively.

**Fig. 2:**
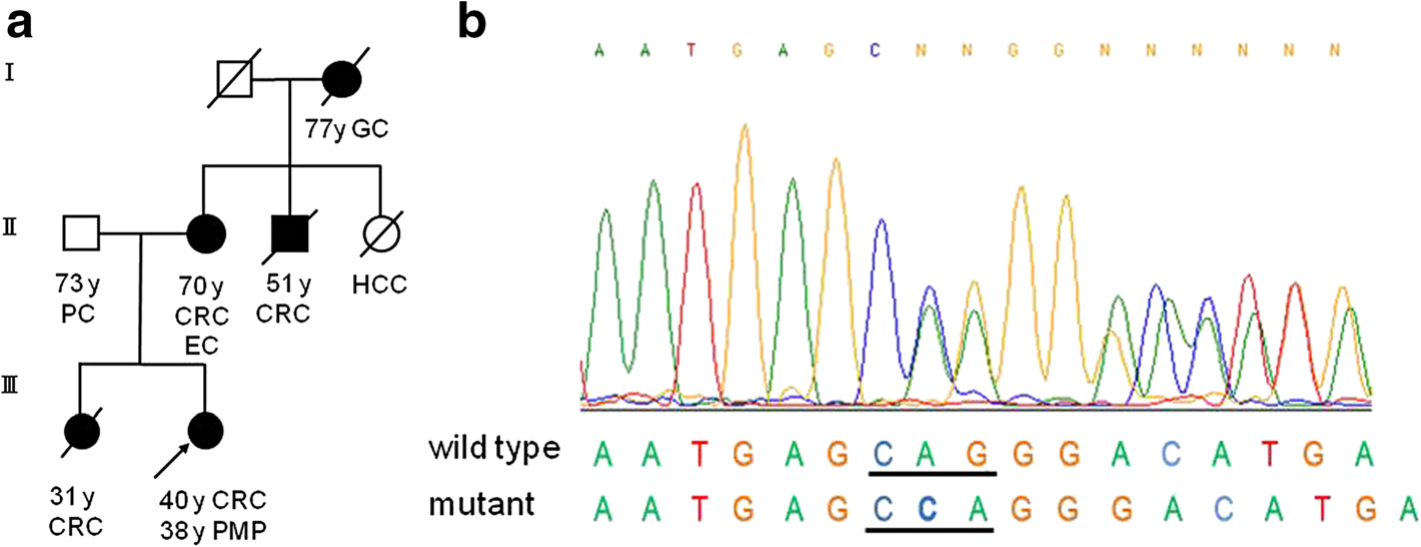
**a** The family tree of the patient. The patient (proband) is indicated by an arrow. Males and females are illustrated by squares and circles, respectively. Unaffected and affected individuals are indicated by open and closed symbols, respectively. Persons deceased are shown by a slash on the symbol. Histories of malignancy and the age of diagnosis are described under the symbols. CRC, colorectal cancer; PC, pancreatic cancer; EC, endometrial cancer; GC, gastric cancer; HCC, hepatocellular carcinoma. **b** Sequence chromatogram containing the mutation (*MLH1* c.1546dupC, p.Q516PfsX3). Codon 516 is underlined

Importantly, the proband’s family history completely fulfilled the Amsterdam criteria (Fig. 2a). The mother suffered from appendiceal cancer, ascending colon cancer, and endometrial cancer, a maternal brother died of rectal cancer at the age of 51, and an older sister of the patient died of sigmoid colon cancer at the age of 31, suggesting the possible diagnosis of LS. After genetic counselling, a written informed consent was obtained from the patient, and a genetic test was performed using DNA from the patient’s peripheral blood. As a result, a germ line mutation consisting of a single nucleotide duplication of C (c.1546dupC, p.Q516PfsX3) in codon516 was identified in the *MLH1* gene (Fig. 2b). This mutation was judged as a deleterious mutation because the duplication resulted in a frameshift mutation inducing a truncation of the MLH1 protein.

**Fig. 3:**
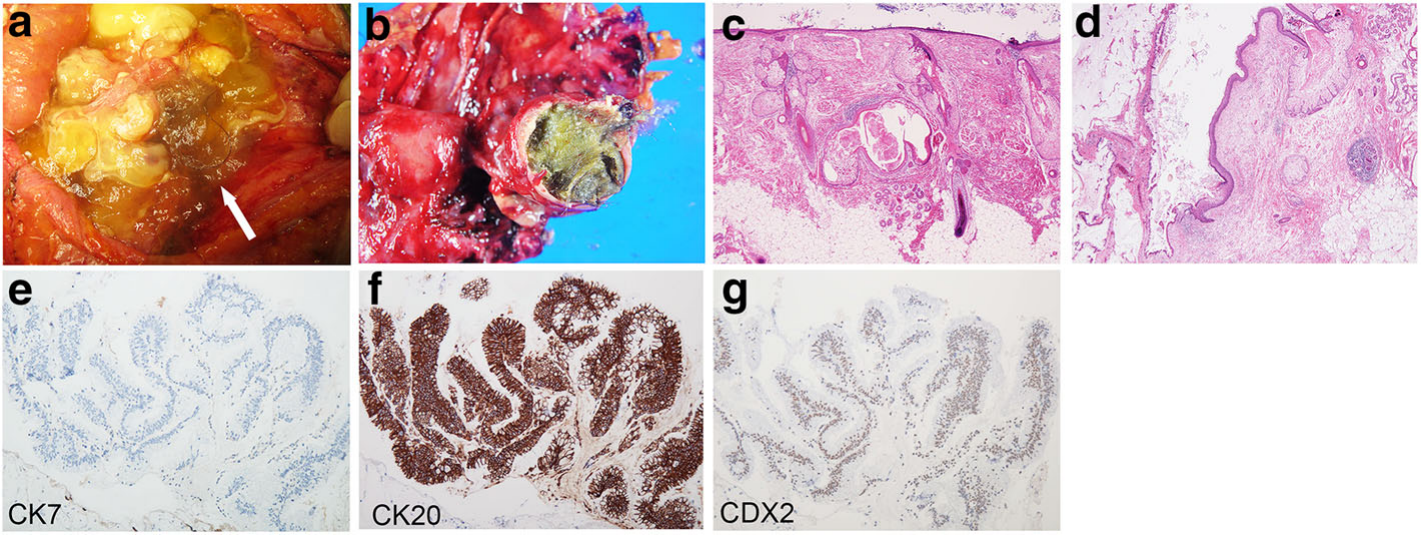
**a** A picture of the patient’s abdominal cavity. Arrow indicates a hair in the abdominal cavity. **b** Macroscopic appearance of the patient’s ovary. **c** Borderline lesion containing epithelium with severe atypia in the teratoma. **d** Histology of the right teratoma containing various differentiations such as squamous epithelium, hair follicles, sebaceous materials, and mucinous epithelium. **e**, **f**, and **g** Immunohistochemical staining of the mucinous epithelium with anti-CK7 (**e**), anti-CK20 (**f**), and anti-CDX2 (**g**) antibodies

An open laparotomy was performed, where abundant mucinous ascites mixed with hair was found in the peritoneal cavity (Fig. 3a). In addition, surgical exploration identified a ruptured left ovarian cystic mass of 16 cm in size and a non-ruptured right ovarian cystic mass of 7.5 cm, but the appendix was normal. The patient underwent complete cytoreductive surgery (CRS) including total abdominal hysterectomy with bilateral salpingo-oophorectomy, total peritonectomy, appendicectomy, greater and lesser omentectomy, splenectomy, cholecystectomy, and resection of the liver capsule. Following the CRS, hyperthermic intraperitoneal chemotherapy (HIPEC) was added with mitomycin C at 10 mg/m^2^ for 1 h. The post-operative course was uneventful.

Both ovarian tumours were filled with copious gelatinous fluids containing multiple hairs and yellowish sebaceous materials (Fig. 3b). Histological examination revealed that the right ovarian tumour was a mature cystic teratoma accompanied by an endometriotic cyst. It was reported that 3 to 8% of primary ovarian mucinous tumours are accompanied by teratoma [26–28]. The teratoma-associated mucinous tumours were also described to show various histological appearances such as cystadenomatous, borderline tumour-like to adenocarcinoma, tumours with entity of goblet cell carcinoid, and low grade adenomatous mucinous tumours. Consistently, the left tumour was a mixture of mucinous cystic tumour and teratomatous lesion. Most of the tumour cells showed mild cellular atypia but complex structural changes classifying the tumour into an intermediate malignancy (Fig. 3c). Additionally, a part of the tumour demonstrated stromal invasion, which corresponds to moderately differentiated adenocarcinoma. The teratomatous tumours predominantly consist of ectodermal components such as skin and its adnexa, cartilage, tracheobronchial epithelium, and included mucin-producing epithelium depicting lower intestinal tract (Fig. 3d). Immunohistochemical study revealed that the left ovarian tumour with intermediate malignancy was negative for CK7 (Fig. 3e), and diffusely positive for CK20 and CDX2 suggesting that it has a lower intestinal phenotype (Fig. 3f and g). However, the region of moderately differentiated adenocarcinoma was positive for CK7 and CDX2, and negative for CK20, indicating aberrant cytokeratin staining pattern due to malignant transformation. Histological examination of the peritoneum, omentum and implants found mucinous material and a small amount of tumour cells (pseudomyxoma). It is of note the histology of the appendix was normal. These data suggested that the pseudomyxoma peritonei may result from mucin-producing epithelial cells derived from ovarian teratoma.

**Fig. 4:**
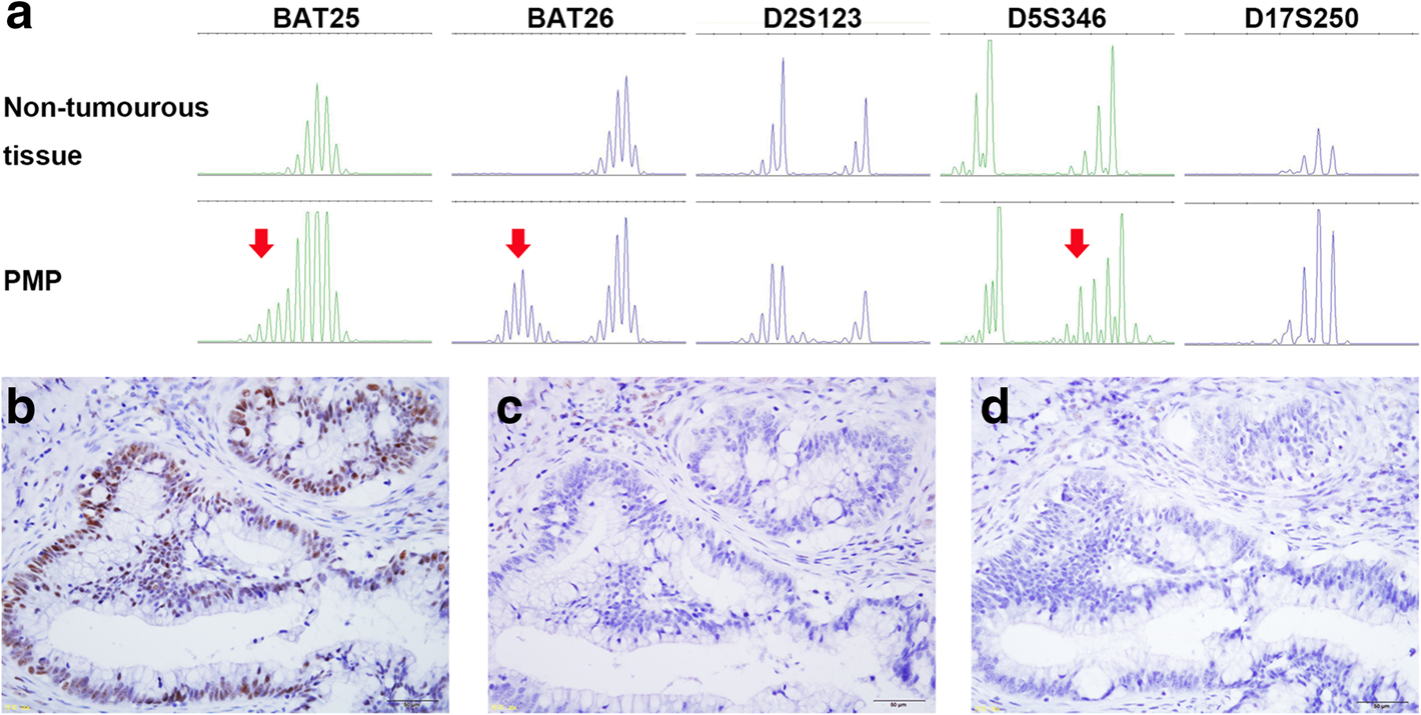
**a** Microsatellite analysis of the PMP. Arrows depict aberrant peaks in tumorous tissues compared with non-tumorous tissues indicating microsatellite instability-high. **b**-**d** Immunohistochemical staining of mismatch repair proteins using anti-MSH2 (**b**), anti-MLH1 (**c**), and anti-PMS2 (**d**) antibodies

To clarify the aetiology of the ovarian tumour, we carried out microsatellite instability (MSI) analysis with the Bethesda panel (D2S123, D5S346, D17S250, BAT25, and BAT26). Interestingly, the DNA from the left ovarian tumour was positive for MSI in three of the five markers (Fig. 4a) indicating high microsatellite instability (MSI-high). Additional immunohistochemical analysis showed positive staining for MSH2 and negative for MLH1 and PMS2 (Fig. 4b-d), which is in complete agreement with the germ line mutation in *MLH1*. These data suggested that the deficiency of mismatch repair system was associated with the ovarian tumour.

After the surgery for PMP, the patient was enrolled in a surveillance programme of PMP. In the 2-yearly colonoscopy, a transverse colon cancer of 2.5 cm in size was identified. The patient underwent right hemicolectomy where no recurrence of PMP was detected. Histological examination of the tumour revealed tubular adenocarcinoma of the colon with an UICC stage of pT2N0M0 (data not shown).

Since our case underwent complete CRS and HIPEC for PMP and curative operation for transverse colon cancer, a good prognosis of the patient is expected. However, if recurrent disease may appear in the patient, we may need to consider immune checkpoint inhibitors for the treatment, because mismatch repair-deficient tumours are more effective to anti-programmed death 1 (PD1) monoclonal antibodies [29].

## Conclusion

So far three groups have reported six PMPs derived from ovarian mature cystic teratoma [3, 25, 30]. In the papers, data of immunohistochemical staining are shown for six tumours. One of the six was partly CK7+, and diffuse CK20+ and diffuse CDX2+ [25], the second case was partly CK7+, and diffuse CK20+ and partly CDX2+ [25], the third to fifth cases were all CK7- and diffuse CK20+ [3], and the sixth case was CK7+, CK20+, and CDX2+ [30]. These data may imply that both lower-gastrointestinal tract type mucinous tumours and primary ovarian mucinous tumours developed in the ovarian teratoma may become the origin of PMPs. In this case, the patient’s teratoma contained various types of epithelium, and the majority of cystic lesion was shown to be lined with mucinous epithelium similar to lower intestinal tract by histological and immunological examination (CK7-/CK20+/CDX2+). In addition, the epithelial cells showed from benign to malignant transformation. The region of moderately differentiated adenocarcinoma in the cystic teratoma with the positive immunohistochemical staining for CK7, strongly suggested that the PMP should have originated from the primary ovarian mucinous epithelium in the teratoma.

This is the first report of PMP originating from an ovarian mature teratoma in a Lynch syndrome patient. Two reports of cystic teratoma in Lynch syndrome have been archived in public databases. One of the two is a case in a Taiwanese LS family [30], and the other is a case of Muir-Torre syndrome, a subtype of Lynch syndrome involving a combination of sebaceous neoplasms of the skin and internal malignancies [31]. However, these two teratoma cases did not accompany PMP. Importantly, the latter case showed loss of MSH2 expression in the sebaceous adenoma within mature cystic ovarian teratoma suggesting the involvement of mismatch repair deficiency. Although the genetic changes associated with teratogenesis have not been clarified, impaired mismatch repair machinery might result in the genetic changes associated with the teratomas.

In conclusion, mismatch repair defect may cause PMP through the development of ovarian tumour, one of the extra-colorectal tumours associated with Lynch syndrome. Further studies will clarify the molecular mechanisms underlying PMP originating from ovarian teratoma.

## Abbreviations

CRC: Colorectal cancer
CRS: Cytoreductive surgery
HIPEC: Hyperthermic intraperitoneal chemotherapy
HNPCC: Hereditary non-polyposis colorectal cancer
LS: Lynch syndrome
*MLH1*: Human mutL homolog 1
MMR: Mismatch repair
*MSH2*: Human mutS homolog 2
*MSH6*: Human mutS homolog 6
MSI: Microsatellite instability
PD1: Programmed death 1
PMP: Pseudomyxoma peritonei
*PMS2*: Postmeiotic segregation increased 2
